# A Study on Consumers’ Willingness to Pay for Remanufactured Products: A Study Based on Hierarchical Regression Method

**DOI:** 10.3389/fpsyg.2019.02044

**Published:** 2019-09-18

**Authors:** Yao Chen, Jinfei Wang, Yinglei Yu

**Affiliations:** ^1^Management School, Shanghai University of International Business and Economics, Shanghai, China; ^2^Antai School of Economics and Management, Shanghai Jiao Tong University, Shanghai, China

**Keywords:** remanufactured product, hierarchical regression method, consumers’ willingness to pay, population variables, product perception

## Abstract

As one of the low-carbon products, remanufactured products are being paid more attention in more and more countries. But the low willingness of Chinese consumers to pay for them makes it difficult for remanufactured products companies to move forward in the Chinese market. This study explores the factors that affect consumers’ willingness to pay for remanufactured goods, through the hierarchical linear regression method, based on questionnaires. The results show that demographic variables (age, education, occupation, and income), individual subjective variables (environmental awareness, secondhand preferences, and Chinese quality trust) and product perception variables (MP4 quality perceived risk, face risk, and product impact on the environment) have a significant impact on the willingness to pay for remanufactured goods.

## Introduction

In order to save resources, protect the environment and achieve sustainable development, governments, manufacturers and consumers all over the world pay more and more attention to the recycling and reuse of waste products. The concept of circular economy has been paid more and more attention all over the world. The development of remanufacturing industry is one of the important means to realize circular economy and improve the reuse of resources.

Remanufacturing is a technical means of product reuse, which takes the scrap products that reach their service life as raw materials. Through the steps include disassembly, cleaning, classification, testing, repairing, reassembly remanufactured products can be produced with the same quality as new products (Xu, 2007). It is a green and low-carbon means of production, with the production cost of remanufactured products being only about 50% of that of new products, saving 60% of the energy cost and 70% of material cost, almost having no solid waste, and the emission of air pollutants is reduced by more than 80%. Remanufacturing has a history of more than 50 years in developed countries like Europe and the United States, and has formed a large-scale industry. As early as 2005, the global industrial remanufacturing output value has exceeded 100 billion US dollars, and the scale of American remanufacturing industry has reached 75 billion US dollars.

However, in China, remanufacturing industry is still in the initial stage. In March 2008, the National Development and Reform Commission approved 14 enterprises across the country as “pilot enterprises in the auto parts remanufacturing industry” (Xu, 2013). In 2009, the Ministry of Industry and Information Technology identified the first batch of mechanical products remanufacturing pilot units, opened the prelude to the development of China’s remanufacturing industry. Judging from the situation of the automobile industry, by the end of 2017, the number of cars and motor vehicles in China had reached 217 million and 310 million, respectively. Production of automobile in China had entered a rapid growth from 2008, and would reach the peak of automobile scrapping. The theoretical scrapping volume would be more than 7 million in 2017, and the number was expected to reach 13 million in 2019. In fact, the number of scrapped vehicles recovered in China is far lower than this figure. According to the statistics of the Ministry of Commerce, in 2017, there were 1.741 million scrapped motor vehicles in China, and 1.472 million scrapped vehicles accounted for only 0.68 percent of the total number of cars, while the number in Japan is 7%, also far less than the material recovery rate of scrapped vehicles in developed countries (80%) (Institute of Internet Industry, Tsinghua University, 2018).

There are many reasons for the difficulties in the development of China’s remanufacturing industry. But the most obvious problem might be that Chinese consumers have a low willingness to buy remanufactured products (Khor and Hazen, 2016; Sun et al., 2018). Chinese consumers know little about remanufacturing, and many consumers subjectively think that “remanufacturing” is a low-quality renovation and are reluctant to buy remanufactured products. The concept of remanufacturing, which has only been widely mentioned in recent years, is still at a relatively unfamiliar stage for the majority of the public, and cannot be distinguished from second-hand products.

Compared with the theoretical modeling researches, the empirical researches on the remanufacturing market problem are less. Recently, a small number of domestic and foreign scholars began to use empirical methods to study the remanufactured consumer purchase behavior. For example, Guide and Li (2010) carried out an online experiment through eBay, concluding that consumers are willing to buy remanufactured products at a lower price than the new products; Using purchase data from eBay, Sobel et al. (2008) concluded that sellers’ credit and product categories play an important role in the price distinction between new and remanufactured products. Liu et al. (2009) investigated college students to study consumers’ awareness of remanufactured products.

Therefore, from the perspective of consumers, this paper will study consumers’ willingness to pay for remanufactured products and the main factors that affect the willingness to pay by means of hierarchical regression, in the way of questionnaire. In order to find effective means and methods to improve the willingness to pay, to provide a theoretical basis for the market development strategies of relevant enterprises and industries, and to promote the healthy development of low-carbon cycle industry in China.

## Materials and Methods

**FIGURE 1:**
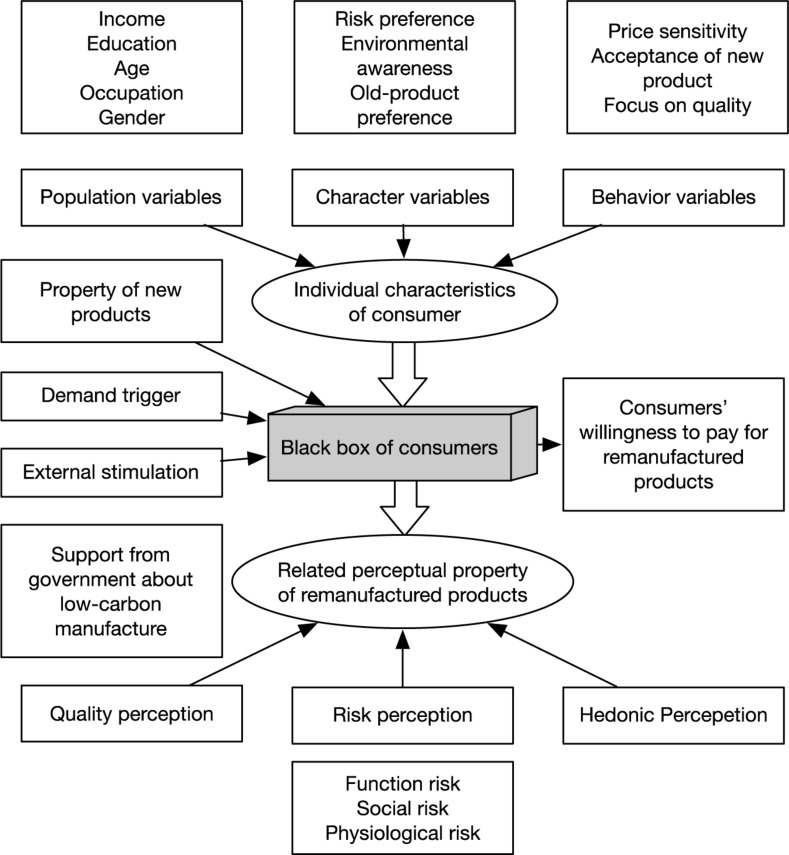
Willingness to pay Model of remanufactured products.

Through discussion, we finally reached a model of consumers’ willingness to pay for remanufactured products (Figure 1). And we designed our questionnaire based on the model and preliminary research.

At the beginning of the questionnaire, the research content was explained in the introduction part. Consent was clearly implied through participation in the study.

### Buying Will

Among many research literatures on remanufacture, it is assumed that different consumers have different willingness to pay, and the willingness to pay for remanufactured goods is lower than the assumption of willingness to pay for new products, that is, if consumers’ willingness to pay for new products is θ, then their buying will for remanufactured products is (1−δ)θ, δ is the perceptual discount of consumers (Chen et al., 2015).

### Risk Preference

Since domestic consumers know little about remanufactured products and they don’t trust the quality and safety of those products fully, so consumers may think that the purchase of remanufactured products has a higher risk. For those consumers with risk preference, they may be willing to try to buy remanufactured goods, regardless of the risks caused by remanufactured goods, so they are more willing to pay for remanufactured goods (Apesteguia and Ballester, 2018). For risk-averse consumers, they may not buy remanufactured goods and are less willing to pay.

### Environmental Awareness

Remanufacturing is the main mode of production to realize the recycling and sustainable development of resources, and the main means of green environmental protection. An important contribution of remanufactured products is the protection of the environment (Bittar, 2018). Through the experiment on red roses, it is concluded that consumers’ environmental preferences have a great impact on consumers’ willingness to pay (Michaud and Llerena, 2008, 2011). Therefore, environmentally conscious consumers may prompt them to buy remanufactured goods, so it is assumed that consumers’ environmental preferences can increase consumers’ willingness to pay for remanufactured goods.

### Price Sensitivity

Because of a large number of energies saving and material saving in the production process, the remanufactured products have a certain discount advantage compared with the original products. For consumers who are concerned about discounts, products of the same quality can be obtained at lower prices, which will greatly promote the purchase of remanufactured products (Bakal and Akcali, 2006; Wu, 2012). Therefore, it is assumed that the price sensitivity of consumers will affect the willingness to pay for remanufactured goods.

### Acceptance of New Products

In the field of marketing, new product acceptance is used to measure whether individual consumers will buy or try the product first when a new product is on the shelves, or whether they insist on buying a familiar brand rather than replacing it (Van Weelden et al., 2016). For Chinese consumers, the remanufacturing industry is still in a rising field, and accordingly, remanufacturing products are also emerging products for most consumers, so it is assumed that. Consumer acceptance of new products will affect the willingness to pay for remanufactured goods.

### Focus on Quality

For remanufactured products, although it indicates that the quality of the product is the same as the new product, but from the point of view of educating consumers, the initial stage effect is not ideal. One of the important points is that consumers pay attention to the quality of the situation, but do not believe in the promotion of remanufactured products (Örsdemir et al., 2014). It is therefore assumed that quality concerns will have an impact on the willingness to pay for remanufactured goods.

### Concern About Face Issue

Face issue refer to people’s desire for being recognized by other people in the relationship (Ting-Toomey and Kurogi, 1998). Affected by Confucianism, traditional Chinese people care about their face in a group (Shi et al., 2010; Chen et al., 2019). Face represents not only self-esteem but also dignity of their family, friends and colleagues. Some consumers regard remanufactured products as old products due to their lack of understanding and think that using old products will lose their face, then they will refuse to buy these products. Thus, it is assumed that consumers’ concern about face issue will have an effect on their willingness to pay.

### Quality Perception

The quality of remanufactured products produced by original equipment manufacturer OEM, and independent remanufacturer (IR) is different. The difference of sellers, prices and brands will affect consumers’ perception of the quality of remanufactured products (Abbey et al., 2015). Different perceptions of quality may also affect consumers’ willingness to pay for remanufactured goods (Sun et al., 2018). Assuming that the higher the consumer perception of the quality of the remanufactured product, the higher the willingness r to pay of the consume for the remanufactured product.

### Reliance on Made in China

With the outbreak of the product quality crisis in China, consumers’ trust in the overall environment of made in China is decreasing day by day (Schniederjans et al., 2011; Franco and Roach, 2018). As a new product that is unfamiliar to the public, remanufactured products will face greater quality trust challenges. Therefore, it is assumed that the environment of trust in the quality of Chinese products will have an impact on the willingness to pay for remanufactured products.

### Old-Product Preference

Remanufactured products are products of the same quality as new products, which are formed after a series of steps, such as disassembly, cleaning, classification, testing, repair, reassembly, etc., but this is obviously different from the new product. To some extent, remanufactured products are “new” products between new and second-hand products. As a result, consumers with old preferences are likely to care about the “old and new” degree of remanufactured goods, and consumers with new preferences will think that remanufactured products are not new products, so they are less willing to pay for remanufactured goods. Consumers with a preference for used goods may be more willing to pay for remanufactured goods. Therefore, we assume that consumers’ preferences for used goods can improve consumers’ willingness to pay for remanufactured goods. Because this concept has no specific definition and historical measurement scale in psychology and marketing, this paper designs a series of scales according to the feedback of consumer opinions in the discussion.

### Product Risk

Consumers take into account the risks of the product itself when evaluating product attributes. From the perspective of marketing theory, product risk can be divided into personal risk, functional risk, social risk, environmental risk and so on (Stone and Grønhaug, 1993). For remanufactured products, in the initial research, consumers showed concern about the realization of their functions, at the same time, there is an impact on their own face and the harm of remanufactured products to their own health (Abbey et al., 2017). The specific contents of the questionnaire can be found in the Supplementary Appendix.

## Results

Data were collected by online research. The platform is www.nquestion.com. Research is carried out during October in 2011. Total number of questionnaires is 1785, among which the number of valid questionnaires is 1106, the validity being 61.96%.

### Reliability and Validity Analysis

As is shown in Table 1, the KMO of the data is 0.670, higher than the value of test questionnaire. The significance level of Bartlett’s Test is 0.000, which means the data was suitable for factor analysis. The result of factor analysis in Table 2 shows that all the variables in multidimensional scale fall into corresponding factors. Factor 1 is environmental awareness, factor 2 is perceived quality, factor 3 is trust on Chinese quality, factor 4 is face issue, factor 5 is price sensitivity, factor 6 is focus on quality, factor 7 is acceptance of new product, factor 8 is old-product preference, factor 9 is Hedonic quality of MP4.

**TABLE 1:** KMO and Bartlett’s test.

Kaiser-Meyer-Olkin Measure of Sampling Adequacy.		0.670
	Approx. Chi-Square	1948.920
Bartlett’s Test of Sphericity df		630
	Sig.	0

**TABLE 2:** Result of factor analysis.

	**Factor**								
	**1**	**2**	**3**	**4**	**5**	**6**	**7**	**8**	**9**
Environmental awareness 1	**0.778**	0.189	0.054	0.052	0.199	–0.072	0.099	0.015	–0.07
Environmental awareness 2	**0.818**	–0.078	–0.07	0.157	–0.055	0	–0.032	–0.036	0.108
Environmental awareness 3	**0.725**	0.095	0.063	0.02	0.095	–0.028	0.118	–0.129	0.051
Environmental awareness 4	**0.685**	0.063	–0.015	–0.138	0.034	0.184	0.009	0.165	–0.019
Environmental awareness 5	**0.859**	–0.039	–0.055	–0.009	–0.068	0.176	0.026	0.113	0.054
Environmental awareness 6	**0.797**	0.083	–0.005	–0.033	0.127	0.151	–0.04	0.022	–0.058
Price sensitivity 1	0.051	0.027	0.007	0.127	**0.746**	0.057	0.191	–0.141	0.014
Price sensitivity 2	0.121	–0.098	–0.085	0.084	**0.86**	0.165	–0.081	–0.014	–0.003
Price sensitivity 3	0.162	–0.102	–0.031	0.041	**0.697**	0.266	0.149	0.305	–0.032
Acceptance of new product 1	0.097	–0.003	–0.211	–0.067	0.23	0.37	**0.55**	–0.102	0.18
Acceptance of new product 2	0.067	0.268	–0.124	0.073	0.186	0.042	**0.799**	–0.012	0.033
Acceptance of new product 3	0.198	–0.323	0.096	–0.234	–0.294	0.224	**0.53**	–0.052	–0.115
Acceptance of new product 4	0.02	–0.002	0.064	0.175	0.123	0.659	**0.454**	–0.087	–0.053
Focus on quality 1	0.222	–0.103	–0.051	0.042	0.108	**0.535**	0.024	–0.012	0.087
Focus on quality 2	0.174	0.065	0.044	0.132	0.24	**0.8**	0.072	–0.128	0.004
Focus on quality 3	0.124	0.257	–0.269	0.001	0.069	**0.434**	–0.287	–0.129	–0.01
Face issue 1	–0.043	0.077	–0.209	**0.748**	–0.069	0.295	0.031	–0.026	0.025
Face issue 2	0.024	0.042	0.087	**0.867**	0.178	0.039	–0.014	0.094	0.002
Face issue 3	0.044	–0.081	0.05	**0.87**	0.114	–0.02	–0.018	0.044	0.026
Trust on quality 1	–0.105	0.181	**0.793**	0.028	0.066	0.096	–0.146	0.103	0.05
Trust on quality 2	–0.029	0.183	**0.865**	0.025	0.004	0.019	–0.118	0.057	0.059
Trust on quality 3	0.135	0.113	**0.648**	–0.055	–0.097	–0.148	0.114	0.014	0.109
Trust on quality 4	–0.061	0.328	**0.703**	–0.062	–0.114	–0.011	0.027	0.041	0.052
Old-product preference 3	0.069	–0.096	0.163	0.212	–0.015	–0.1	–0.052	**0.749**	0.034
Old-product preference 4	–0.015	0.249	–0.161	–0.073	0.007	0.017	–0.119	**0.397**	–0.014
Old-product preference 5	0.044	0.09	0.076	–0.053	0.017	–0.126	–0.041	**0.837**	–0.06
Quality of MP4 1	–0.03	**0.772**	0.057	–0.086	0.029	–0.072	0.121	–0.027	0.021
Quality of MP4 2	0.002	**0.765**	0.068	–0.077	–0.113	–0.009	0.16	–0.047	–0.014
Quality of MP4 3	0.013	**0.795**	0.107	–0.076	–0.029	0.005	0.091	–0.017	0.089
Hedonic quality of MP4 1	0.041	0	0.178	0.035	0.055	–0.056	0.021	–0.083	**0.949**
Hedonic quality of MP4 2	0.037	0.134	0.114	0.035	–0.137	0.226	0.087	0.13	**0.619**
Explained variance ratio	11.154	10.332	8.113	6.705	6.231	6.094	5.115	4.794	3.907

*Bold values shows the composition of factors.*

Through the reliability test of different factors in the factor analysis, we can know that Cronbach’s coefficient alpha is above 0.6, which shows great reliability of questionnaire (Table 3).

**TABLE 3:** Cronbach’s coefficient alpha of test scale.

**Variable**	**Measurement**	**Cronbach’s α**
Environmental awareness	Environmental awareness 1	0.898
	Environmental awareness 2	
	Environmental awareness 3	
	Environmental awareness 4	
	Environmental awareness 5	
	Environmental awareness 6	
reliance on Chinese quality	Reliance on Chinese quality 1	0.799
	Reliance on Chinese quality 2	
	Reliance on Chinese quality 3	
	Reliance on Chinese quality 4	
Quality perception of MP4	Quality perception of MP4 1	0.901
	Quality perception of MP4 2	
	Quality perception of MP4 3	
Face issue	Face issue 1	0.837
	Face issue 2	
	Face issue 3	
Acceptance of new product	Acceptance of new product 1	0.674
	Acceptance of new product 2	
	Acceptance of new product 4	
old-product preference	Old-product preference 3	0.698
	Old-product preference 4	
	Old-product preference 5	
Price sensitivity	Price sensitivity 1	0.834
	Price sensitivity 2	
	Price sensitivity 3	
Hedonic quality of MP4	Hedonic quality of MP4 1	0.746
	Hedonic quality of MP4 2	

From the result, it is obvious that all standardized factor loadings exceed 0.6. It means that the scale has a high convergent validity. According to each scale of factor analysis, Pearson correlation coefficient matrix of these scales could be calculated (Table 4). All scales are less than moderate related, which shows high discriminant validity.

**TABLE 4:** Pearson correlation coefficient matrix of each factor.

	**Environmental awareness**	**Price sensitivity**	**Acceptance of new products**	**Face issue**	**Quality perception of MP4**	**Hedonic quality of MP4**	**Reliance on Chinese quality**	**Old-product preference**
Environmental awareness	1	0.338^∗∗^	0.292^∗∗^	0.195^∗∗^	0.133^∗∗^	0.063^∗^	0.114^∗∗^	0.155^∗∗^
Price sensitivity	0.338^∗∗^	1	0.423^∗∗^	0.194^∗∗^	0.013	0.060^∗^	0.010	0.126^∗∗^
Acceptance of new products	0.292^∗∗^	0.423^∗∗^	1	0.181^∗∗^	–0.012	0.072^∗^	–0.040	–0.002
Face issue	0.195^∗∗^	0.194^∗∗^	0.181^∗∗^	1	0.136^∗∗^	0.153^∗∗^	0.126^∗∗^	–0.089^∗∗^
Quality perception of MP4	0.133^∗∗^	0.013	–0.012	0.136^∗∗^	1	0.050	0.376^∗∗^	0.123^∗∗^
Hedonic quality of MP4	0.063^∗^	0.060^∗^	0.072^∗^	0.153^∗∗^	0.050	1	0.028	–0.058
Reliance on Chinese quality	0.114^∗∗^	0.010	–0.040	0.126^∗∗^	0.376^∗∗^	0.028	1	0.137^∗∗^
Old-product preference	0.155^∗∗^	0.126^∗∗^	–0.002	–0.089^∗∗^	0.123^∗∗^	–0.058	0.137^∗∗^	1

*^∗∗^Correlation is significant in the level of 0.01 (double-tail). ^∗^Correlation is significant in the level of 0.05 (double-tail).*

The convergent validity is represented by the relationship between each question and the total score of each scale. As is shown in Table 5, the internal correlation coefficients all exceed 0.6. It means the scale has high convergent validity.

**TABLE 5:** Internal correlation coefficient of test scale.

**Variable**	**Measurement**	**Correlation coefficient**	**Variable**	**Measurement**	**Correlation coefficient**
Price sensitivity	Price sensitivity 1	0.826^∗∗^	Acceptance of new products	Acceptance of new products 1	0.781^∗∗^
	Price sensitivity 2	0.897^∗∗^		Acceptance of new products 2	0.797^∗∗^
	Price sensitivity 3	0.876^∗∗^		Acceptance of new products 4	0.757^∗∗^
Reliance on Chinese quality	Reliance on Chinese quality 1	0.888^∗∗^	Hedonic quality of MP4	Hedonic quality of MP4 1	0.900^∗∗^
	Reliance on Chinese quality 2	0.910^∗∗^		Hedonic quality of MP4	0.887^∗∗^
	Reliance on Chinese quality 3	0.517^∗∗^	Face issue	Face issue 1	0.770^∗∗^
	Reliance on Chinese quality 4	0.842^∗∗^		Face issue 2	0.924^∗∗^
Environmental awareness	Environmental awareness 1	0.778^∗∗^		Face issue 3	0.904^∗∗^
	Environmental awareness 2	0.799^∗∗^	Quality perception of MP4	Quality perception of MP4 1	0.911^∗∗^
	Environmental awareness 3	0.805^∗∗^		Quality perception of MP4 2	0.917^∗∗^
	Environmental awareness 4	0.825^∗∗^		Quality perception of MP4	0.915^∗∗^
	Environmental awareness 5	0.850^∗∗^	Old-product preference	Old-product preference 3	0.664^∗∗^
	Environmental awareness 6	0.829^∗∗^		Old-product preference 4	0.672^∗∗^
				Old-product preference 5	0.734^∗∗^

### Hierarchical Regression

The reasons to use hierarchical regression are as follows. Firstly, a hierarchical relationship exists among the factors that affecting consumers’ willingness to pay. For example, population variables such as gender, age, occupation will affect individual subjective factors like face issue, old-product preference and environmental awareness. But there is no reverse effect. Likewise, individual subjective factors have an influence on product perception (such as quality of MP4, the healthy effect of MP4). There is no reverse effect either. Thirdly, the degree of different factors contributing to the willingness to pay could be seen by the change of *R*^2^ in the hierarchical regression. In general, the research will be carried out based on multiple liner regression, using three levels, including population variable in model 1, individual subjective factors in model 2 and product perceptions in model 3.

First of all, this paper makes a regression analysis of the demographic variables (Table 6), and brings the age, sex, education level, income, occupation and geographical variables into the regression equation, considering that the region and occupation asked in the questionnaire are nominal variables. So, we set it as a dummy variable to ensure the accuracy of the study. By stepwise regression, it was found that the age, teacher/professor and education level of the interviewees were positively correlated with the willingness to pay for the remanufactured MP4. According to the idea of hierarchical regression, when the income variable is put into the regression equation, the correlation between age and willingness to pay for MP4 decreases significantly, indicating that the effect of income on willingness to pay for MP4 can be explained by age to some extent. This is also in theory in line with our understanding.

**TABLE 6:** Regression coefficient of population variables.

	**Model 1**	**Model 2**
Age	0.079^∗∗^	0.061^†^
Occupation5	0.081^∗∗^	0.079^∗∗^
Education	0.096^∗∗^	0.089^∗∗^
Income		0.048^†^
Adjusted *R*^2^	0.021	0.026
F	8.711^∗∗∗^	9.703^∗∗∗^

*^†^*P* < 0.1. ^∗^*P* < 0.05. ^∗∗^*P* < 0.01. ^∗∗∗^*P* < 0.001. *^∗^Occupation5* is dummy variable, *Occupation5* = 1 represents the interviewee is professor or teacher, *Occupation5* = 0 represents interviewee in other occupation.*

The results indicated that people’s willingness to pay for remanufactured MP4 is dependent on age, occupation, education and income. There is a positive correlation between age and willingness to pay, that is, the older the age, the stronger the willingness to pay. Teachers/professors are willing to pay more for the remanufactured MP4 than other occupations, and the higher the level of education, the stronger the willingness to pay for the remanufactured MP4. The higher the income, the stronger the willingness to pay for MP4. Other demographic variables were not significantly related to the willingness to pay for remanufactured MP4.

Given the impact of demographic factors on willingness to pay, the following analysis incorporates them into control variables to further analyze the impact of other factors on willingness to pay for remanufactured products. Bring a variety of predictive variables that may affect willingness to pay into the regression equation, and the results are shown in the figure below. In model 2 (Table 7), the preferences for new or old, environmental awareness and quality trust in China were significantly correlated with the willingness to pay for remanufactured MP4, while others had little impact on the results. At the same time, it can be found that after the addition of individual subjective factors, the relationship between demographic variables and MP4 willingness to pay still exists, showing the robustness of the results.

**TABLE 7:** Regression coefficient of Consumers’ subjective variables.

	**Model 1**	**Model 2**
Population Variables		
Age	0.061^†^	0.059^†^
Occupation5 (dummy variable)	0.079^∗∗^	0.079^∗∗^
Education	0.089^∗∗^	0.089^∗∗^
Income	0.048^†^	0.038
Individual Subjective Variable		
Face issue		0.039
Old-product Preference		0.105^∗∗∗^
Environmental Awareness		0.096^∗∗^
Price sensitivity		−0.004
Acceptance of new Products		0.026
Focus on quality		−0.061
Reliance on Chinese Quality		0.070^∗^
Adjusted *R*^2^	0.026	0.059
F	9.703^∗∗∗^	11.044^∗∗∗^

*^†^*P* < 0.1, ^∗^*P* < 0.05, ^∗∗^*P* < 0.01, ^∗∗∗^*P* < 0.001.*

According to the results above, we will add quality perception variables, while keeping variable that have a significant effect, to do regression (Table 8). It is obvious that face issue risk, quality risk, environmental risk, functional ability and purchasing willingness have positive significant correlations. Based on the change from model 2 to model 3, adding product perception variables helps explain the model and the result still has a high robustness.

**TABLE 8:** Regression coefficient of Consumers’ quality perception.

	**Model 1**	**Model 2**	**Model 3**
**Population Variables**			
Age	0.061^†^	0.059^†^	0.059^†^
Occupation5 (dummy variable)	0.079^∗∗^	0.079^∗∗^	0.072^∗∗^
Education	0.089^∗∗^	0.089^∗∗^	0.097^∗∗^
Income	0.048^†^	0.038	0.032
**Individual Subjective variable**			
Old-product preference		0.105^∗∗∗^	0.086^∗∗^
Environmental awareness		0.096^∗∗^	0.081^∗∗^
Reliance on Chinese product		0.070^∗^	0.051^†^
**Product Perception**			
Healthy risk of MP4			−0.041
Face issue of MP4			0.061^†^
Quality risk of MP4			−0.114^∗∗∗^
Functional ability of MP4			0.055^†^
Environmental effect of MP4			0.068^∗^
**Adjusted *R*^2^**	**0.026**	**0.059**	**0.074**
**F**	**9.703^∗∗∗^**	**11.044^∗∗∗^**	**13.896^∗∗∗^**

*Bold values shows the composition of factors. ^†^*P* < 0.1, ^∗^*P* < 0.05, ^∗∗^*P* < 0.01, ^∗∗∗^*P* < 0.001.*

Based on the above analysis, we understand the influencing factors of consumers’ willingness to pay for remanufactured MP4 products as a whole. The model concluded is that:

Willingnes to pay for remanufactures MP4
  = 0.059 × Age + 0.072 × Occupation + 0.097 × Education
  + 0.032 × Income + 0.086 × Old − product preference
  + 0.081 × Environmantal awareness + 0.051 × Reliance on Chinese quality
  + 0.061 ^∗^ MP4 Face issue − 0.114 × MP4 Quality risk + 0.055
  ^∗^ MP4 Functional ability + 0.068 × environmental effect

## Discussion

According to the results of the investigation, it is found that the main factors that determine the willingness to pay for remanufactured MP4 are consumer’s age, job, education, consumer’s preferences for new or old, environmental awareness, reliance on Chinese quality, and perceived MP4 face risk, quality risk, the function of hedonism and the impact of remanufactured MP4 on the environment.

There are the following discussions:

*The higher the consumer’s preference for old models is, the higher his or her willingness to pay for the remanufactured products is*. For the consumers who have preferences for old models, as long as the product does not affect its basic using, they are willing to pay more for remanufactured products.
*The higher the consumer’s environmental awareness is, the higher his or her willingness to pay for the remanufactured products is*. A consumer with a high awareness of environmental protection will take the influence of the products into consideration. Thus, they would like to pay more for them.
*The more the consumer have reliance on Chinese quality, the higher his or her willingness to pay for the remanufactured products is*. Because of the improvement of Chinese quality (Made in China), an increasing number of consumers start to trust in these products.
*The riskier the consumer perceives the quality of remanufacturing products, the lower his or her willingness to pay for the remanufactured products is*. This is our common sense that when the products have a certain amount of uncertainties, consumers will not purchase them.
*The higher level of awareness of consumers of the remanufacture industry’s riskiness is, the lower his or her willingness to pay for the remanufactured products is*. If the remanufacturing products will cause environmental damages, the consumers will not tend to purchase them and vice versa.
*Due to some factors, different groups of people have different levels of willingness to pay for remanufactured products*. From the regression analysis, we found that age, work, education and income can affect people’s willingness to pay for the remanufactured MP4. Elder consumers, consumers with higher education level, consumers with higher income will tend to pay for remanufactured products. Theoretically, it is not hard to find the positive correlation between consumers’ income and willingness to pay. For age, job position, and education, we can perceive as consumers who are elder and with higher level of education and better job are more rational so that their acceptance of remanufactured products is higher.

Based on the above conclusions, we believe that in order to improve consumers’ recognition and willingness to pay for remanufactured products, the following points should be done:

(1) Advertising and other media methods can enhance consumers’ understanding of remanufacturing products. It is not only necessary to make people know the existence of remanufactured products, but also to present the whole production process of remanufactured products to people, so as to form a rational understanding and tell consumers the advantages of these products such as environment-friendly, energy-efficiency, and high-quality, etc.
(2) The government should introduce relevant policies to protect the rights and interests of consumers to buy remanufactured products and build up consumers’ confidence in remanufactured products. In addition, improve the overall quality of Chinese products, so that consumers have a better impression of Chinese-made products, so that consumers do not have to worry about buying Chinese-made remanufactured products.
(3) Remanufacturing enterprises could consider selling remanufactured products in the second-hand market, such as Taobao, as their marketing channel in which consumers tend to be more receptive to remanufactured products.
(4) The publicity of remanufactured products should reflect the advantages of remanufactured products, such as environmental protection, quality. These factors show a significant correlation to the willingness to pay in the study.

There are still some further studies could do. On one hand, the factors that affect the willingness to pay could be expanded, for example, some objective factors, such as region (western region and eastern coastal area), purchase experience, knowledge of remanufactured products (Wang and Hazen, 2016) understanding of remanufactured products, and so on, which may be the main reason why the interpretation of the model is not high. On the other hand, product categories have a certain impact on the expansion of the remanufacturing market. This paper examines that MP4 belongs to hedonic products, which can deeply investigate the corresponding effects of general functional products and luxury categories.

## Data Availability

The raw data supporting the conclusions of this manuscript will be made available by the authors, without undue reservation, to any qualified researcher.

## Ethics Statement

Ethical review and approval were not required for the study on human participants in accordance with the local legislation and institutional requirements. Informed consent was inferred through the completion of the study.

## Author Contributions

YC holds the structure of the manuscript. YY collated the manuscript. JW revised the manuscript.

## Conflict of Interest Statement

The authors declare that the research was conducted in the absence of any commercial or financial relationships that could be construed as a potential conflict of interest.

## Supplementary Material

APPENDIX Data and the output.
